# Ophthalmology

**Published:** 1892-07

**Authors:** Alvin A. Hubbell


### Progress in Medical Science.

### OPHTHALMOLOGY.

By ALVIN A. HUBBELL, M. D.

### AN INSTRUMENT FOR THE DETERMINATION OF CONVERGENCE, POWER AND THE POSITION OF REST OF THE EYES.

M. STRAUB, of Utricht, *Ophthalmic Review*, April, 1892, describes an instrument for rapidly testing the latent position of the eyes in distant and near fixation, which consists of an oblong mirror, sixteen by four centimeters, attached to a narrow ribbon, furnished with a centimeter scale. The ribbon is about one and a half meters in length, and has a black rule fastened to one end. It is applied as follows : A small ink-spot is made on the bridge of the patient's nose between the eyebrows, and he is seated opposite to the observer with his back to the window. The observer holds the mirror with the left hand against his own forehead, with its long axis horizontally, so that the patient can look in it. The mirror must then be turned on its horizontal axis, enabling the patient to see the ink-spot on his own nose, or in a subsequent observation some distant object behind him which has been pointed out to him before taking his seat. This arrangement enables the observer, whilst the patient is accommodating his eyes for different distances, to make the usual test for squint by means of the ruler, held in the right hand, and used alternately to cover the right and left eye. Meanwhile, the ribbon placed behind the patient's ear measures the distance from the eye to the looking-glass, *i. e.*, half the distance for which the eye is accommodated, if the ink-spot is looked at.

Each eye is covered and uncovered frequently. The patient will sometimes keep on converging at first, and only after several coverings and uncoverings does the innervation of the covered eye give way, and the eye enter into what might be termed the relative position of rest. The relative position of rest is determined at three distances: six meters or more, fifty centimeters, and thirty centimeters, and it is noted whether there is convergence or divergence, and whether the angle of convergence or divergence be large or small.

The determination, thus, of the position of rest at various distances, shows that in viewing a near object convergence does not continue unless required, and the covering up of one eye would normally cause relative divergence. At a distance of thirty or fifty centimeters, deviation of the covered eye must occur in a very slight degree; considerable deviation indicates want of convergence power, which is clinically important when binocular vision exists. Insufficiency of convergence can thus be excluded or diagnosticated without difficulty.

---

### EXPERIMENTS ON THE LYMPH-STREAMS AND LYMPH-CHANNELS OF THE EYE.

Dr. H. Gifford, of Omaha, Neb. (*Arch. of Ophthalmology*, April, 1892), has given the results of experiments which he has made to determine the paths by which the chambers and larger lymph-spaces of the eye communicate with each other and with the parts external to the eye, and they are so instructive that a brief summary of his paper is hereby submitted to the readers of the Journal.

His experiments have been in progress since 1886, and have been made with various substances and by various methods, upon both living and dead animals. The substances principally used were ferro-cyanide of potassium, fluorescein, pigment, and anthrax bacilli. He describes the methods and conclusions of Memorsky, Knies, Weiss, Ulrich, and Stilling, by which solutions of ferro-cyanide of potassium are introduced into the living eye, the eye enucleated, and the ferro-cyanide precipitated by placing it in a ferric chloride solution, the blue color of the precipitate thus produced showing the tissues into which it has been carried during life. He also studies the effect of the same substance introduced into the dead eye. He concludes, however, that while the blue lines obtained by the ferro-cyanide method are at first sight very striking, and may, to some extent, be influenced by vital processes, there is no evidence to show that they really follow the paths of normal lymph-streams. His experiments with solutions of fluorescein injected subcutaneously, or into the living vitreus, were not convincing.

He believes the pigments gave the most reliable results. He has used injections into the eye, of india-ink and of cinnabar, suspended in one-half to three-quarters per cent. sodium chloride solution. The cinnabar has the advantage of being less likely than india-ink to cause marked reaction, but the particles are heavier and less easily transported by the lymph-streams. India-ink reaches fine channels, which he has not found demonstrable with cinnabar. In injections, *post mortem*, into the anterior chamber and its outlets, he has obtained the most striking results with asphalt chloroform (fifteen per cent.) and a solution of alkanet in turpentine.

Although he is convinced that the introduction of small quantities of pigment into the chambers of the eye and into the cranial cavity in live rabbits, gives the most trustworthy results as to the paths and directions of the lymph-currents, yet there are decided limitations. He has been inclined to believe that wherever considerable pigment was carried into lymph-channels, apparently without the aid of leucocytes, that it must have been transported by some regular stream; but it has been found that in some channels, especially in the finer ones, the pigment finds its way equally well in precisely opposite directions. The judgment of Leber is thus confirmed as to the caution with which conclusions must be drawn from the presence of pigment in any tissue as to the method of its transport.

After detailing his experiments with pigment, and the bacilli of anthrax, Gifford draws the following conclusions :

1. The ferro-cyanide and fluorescein methods are not calculated to give trustworthy results in determining the physiological currents of the eye. Many of the blue lines, obtained by the former method, represent simply the boundaries between tissue into which the ferro-cyanide has become diffused and that containing none. The lines upon which most stress has been laid can be obtained perfectly well in the dead eye.

2. The view of Stilling, that there is no outlet from the vitreus forward around the lens, is incorrect. The zonula (suspensory ligament of the lens) is freely permeable for solid particles, free pigment being carried regularly from the vitreus into the anterior chamber. It is probable that the fluid secreted by the ciliary processes, posterior to the zonula, divides into two portions, one part passing forward into the posterior chamber, and thence through the pupil into the anterior chamber; the other passing back through the vitreus and out through the central canal of the optic nerve into the tissues of the orbit.

3. There is no evidence of any current passing from the posterior chamber through the iris root.

4. There is no evidence of any current from the anterior chamber through the membrane of Descemet into the cornea, although pigment particles may be taken up from the aqueus by the protoplasm of this membrane. As there is shown to be a free connection for non-diffusible substances between Fontana's spaces and the circum-corneal veins, it is probable that the greater part of the aqueous humor leaves the eye in this way. Other finer lymph-channels lead from Fontana's spaces into the posterior layers of the cornea, into the peri-vascular spaces of the sclero-corneal junction (possibly), into the sclera, choroid, and peri-choroidal space. Whenever these channels communicate with spaces in which there is a lower pressure than in the anterior chamber, they must, on the whole, serve, to some extent, as outlets.

5. Between the retinal-pigment epithelium and the layer of rods and cones is a tolerably well-defined space, from which pigment passes freely into the retina, but not into the choroid, except along occasional penetrating blood-vessels in the neighborhood of the optic nerve.

6. Certain facts, such as the regular passage of pigment and bacilli from Fontana's spaces into the cornea, and the progress of sub-conjunctival hemorrhages in the same direction, together with the impermeability of Descemet's membrane from behind, speak for the nourishment of the cornea from its periphery, although the corneal lymph-stream, if it exists, is very weak.

---

### SYMPATHETIC OPHTHALMIA AFTER CATARACT OPERATIONS.

L. de Wecker, of Paris (*Annals d'Oculistique*, February, 1892), says that one disadvantage of excising a portion of the iris in cataract extraction is that the capsule-wound and corneal-wound are left in contact, and the capsule thus becomes entangled in, and united to the corneal cicatrix, whereby irritation is set up in the iris and ciliary body, and sympathetic ophthalmia may follow. Since he has adopted the flap extraction and given up iridectomy, he has not had a single case of sympathetic ophthalmia. During a period of eight years, 1,500 cases were operated upon by a section approximating to that of von Græfe, combined with iridectomy. Among these were five cases of sympathetic ophthalmia. During a subsequent period of twenty-two years, 5,000 flap extractions have been made, with or without iridectomy, with only two cases of sympathetic ophthalmia, which apparently occurred before the adoption of the purely corneal section.

---

### GLAUCOMA AFTER DISCISSION OF SECONDARY CATARACT.

Knapp (*Archives of Ophthalmology*, April, 1892,) records ten cases of glaucoma following discission of the capsule after "simple" extraction of cataract. These constituted a little more than one per cent. of his cases of extraction without iridectomy. As to its pathogenesis, he says he does not venture any hypothesis. One case recovered under the use of eserine ; others did not. A subsequent iridectomy, at least in acute cases, cured the glaucoma. In most cases it was made upwards with a lance-shaped knife, the incision being somewhat back of the one made for the previous extraction, and not very broad. Iris forceps, even those with teeth projecting downward, failed to grasp the iris, and the latter was, therefore, drawn out with the blunt hook and cut off. A little vitreus always protruded, but caused no ill-effect. Dr. Knapp does not feel inclined, because of one per cent. of glaucoma, to return to the combined method of extraction with iridectomy.

---

Idiosyncrasy cannot be too carefully considered, especially in the administration of anesthetics. Cocaine, locally, is considered practically harmless, yet many cases are reported where fatal results have obtained. In the January number of *La Medicine Contemporaire*, M. Terrier reports a case where thirty-eight centigrammes of the salt, injected into the sac of a hydrocele and soon after withdrawn, was followed in an hour by extreme paleness of the face, dilatation of the pupils, foaming of the mouth, general convulsions, and fatal syncope.

In our own experience, seven and one-half grains of chloral, to a vigorous woman of thirty, repeated in an hour, gave anxious hours of unintermitting artificial respiration. . . .—*Kans. Med. Jour.*

